# Editorial: Mechanical ventilation in anesthesia and critical care animal patients, volume II

**DOI:** 10.3389/fvets.2024.1402629

**Published:** 2024-05-02

**Authors:** Aline Magalhães Ambrósio, Denise Tabacchi Fantoni

**Affiliations:** Department of Surgery, Faculty of Veterinary Medicine and Animal Science, University of São Paulo, São Paulo, Brazil

**Keywords:** mechanical ventilation, dogs, horses, alveolar recruitment maneuver (ARM), foal

## Introduction

Mechanical ventilation, a cornerstone of modern veterinary medicine, has evolved significantly. It has become an indispensable tool in ensuring the safety and stability of animals during surgery. It plays a crucial role in intensive care units, where it aids in the recovery of critically ill patients. The intricacies and nuances of mechanical ventilation are often overlooked, yet they hold the key to the successful management of anesthesia and the survival of animals in critical conditions.

In this editorial, we investigate mechanical ventilation during animal anesthesia or intensive care, exploring its significance, advancements, and challenges that veterinarians must weigh. We aim to shed light on this technology's pivotal role in enhancing the quality of care provided to animals and the constant pursuit of refinement to minimize potential risks and optimize outcomes. As the field of veterinary medicine continues to advance, we must maintain a comprehensive understanding of the intricacies of mechanical ventilation. Through this exploration, we aim to underscore the importance of this life-saving technique and inspire a continued commitment to excellence in animal anesthesia and intensive care.

This Research Topic presents four new papers that illuminate these issues in mechanical ventilation in horses and dogs.

## Mechanical ventilation in horses

Horses undergoing general inhalation anesthesia often present complications related to the decubitus position in which they are lying on the operating table. Such complications are related to difficulties in gas exchange due to a decrease in the ventilation/perfusion ratio, pulmonary atelectasis, and a drop in blood pressure. Lung atelectasis in horses is produced mainly due to dorsal or lateral decubitus. In dorsal decubitus, the lungs receive compression from the diaphragm produced by the compression of the abdominal viscera (1). In lateral decubitus, the upper lung compresses the mediastinum and, consequently, the lower lung. Due to the loss of functional areas of the lungs, there is a drop in gas exchange, causing a reduction in the partial pressure of arterial oxygen and an increase in the partial pressure of arterial carbon dioxide, impairing cellular processes. Alveolar recruitment maneuvers (ARMs) reverse atelectasis, and positive end-expiratory pressure (2) keeps the alveoli open. However, they are not free from side effects, including barotrauma, volutrauma, and atelectrauma, and monitoring is essential. The evaluation can be done through imaging tests such as computed tomography (CT) in humans and small animals. However, it is only possible in horses through electrical impedance tomography (3, 4), respiratory mechanics, or arterial oxygenation through blood gas analysis. Therefore, Sacks et al. present a study comparing the ventilation distribution measured by electrical impedance tomography (EIT) in foals under diazepam sedation, postural changes, and continuous positive airway pressure (CPAP). Specific spirometry data and F-shunt calculation were also assessed to support the interpretation of EIT variables. They verified that in healthy foals, diazepam administration did not alter the distribution of ventilation or minute ventilation, and the lateral recumbency results in the collapse of dependent lung areas. The CPAP use in dorsal recumbency foals increases pulmonary pressures and improves ventilation in dependent regions, suggesting improvement of ventilation-perfusion mismatch. These findings will help anesthesiologists and intensivists understand what happens in these animals and how to improve ventilation in sedated and lateral recumbent foals. In adult horses, Brandly et al. studied the flow-controlled expiration technique (FLEX) during anesthesia to reduce PEEP requirement in dorsally recumbent. They observed that FLEX ventilation was associated with a lower PEEP requirement due to a more homogenous lung ventilation distribution during expiration. This lower PEEP requirement led to more stable and improved cardiovascular conditions in horses ventilated with FLEX. This study makes an essential contribution to the anesthesia and ventilation of horses as it presents a strategy to treat intraoperative hypoxemia and protect the lungs using lower PEEP to maintain alveolar recruitment.

## Mechanical ventilation in dogs

In dogs, alveolar recruitment is needed to reverse pulmonary atelectasis. Likewise, ARMs can cause lung damage and can be monitored by CT (5, 6), a gold standard method, in addition to EIT, ventilatory mechanics, and blood gas analysis. The lung protection strategy should also employ low tidal volumes and PEEP. Sanchez et al. studied dogs submitted to a stepwise ARM and monitored lung volume distribution by CT. They verified that the CT showed maximum pulmonary aeration distribution by PEEP titration, which occurred at PEEP 20 cmH_2_O and maintained the lungs normoaerated and without hyperaeration. However, based on the best static compliance and driving pressure associated with the absence of hemodynamic changes, the best PEEP value to keep the alveoli open after ARMs was PEEP from 10 and 5 descending for this study condition. In another randomized clinical trial, Rodrigues et al. studied intraoperative protective mechanical ventilation in dogs based on 8 mL.kg^−1^ tidal volume, recruitment maneuvers, and PEEP. Their results showed the possibility of using volumes smaller than 10 mL.kg in dogs to protect the lungs against injuries caused by excessive volumes during mechanical ventilation. In surgeries lasting up to 1 h, there is no need for ARMs if PEEP is maintained from the beginning at 5 cmH_2_O.

## Conclusion

The four studies in this edition demonstrated that performing recruitment maneuvers and subsequent administration of PEEP to keep the alveoli open is an essential technique for reversing hypoxemia in horses and dogs during anesthesia or ICU. These studies have also demonstrated the importance of monitoring these to avoid lung injuries and hemodynamic dysfunctions.

## Author contributions

AA: Writing – original draft, Writing – review & editing. DF: Writing – review & editing.
